# Dust Filtering in LIDAR Point Clouds Using Deep Learning for Mining Applications

**DOI:** 10.3390/s25206441

**Published:** 2025-10-18

**Authors:** Bruno Cavieres, Nicolás Cruz, Javier Ruiz-del-Solar

**Affiliations:** 1 Department Electrical Engineering, Universidad de Chile, Santiago 837-0451, Chile; bruno.cavieres@ug.uchile.cl; 2 Advanced Mining Technology Center, Universidad de Chile, Santiago 837-0451, Chile; nicolascruz2187@gmail.com

**Keywords:** dustfiltering, LIDAR denoising, deep learning, PointNet

## Abstract

In the domain of mining and mineral processing, LIDAR sensors are employed to obtain precise three-dimensional measurements of the surrounding environment. However, the functionality of these sensors is hindered by the dust produced by mining operations. In order to address this problem, a neural network-based method is proposed. This method is capable of filtering dust measurements in real time from point clouds obtained using LIDARs. The proposed method is trained and validated using real data, yielding results that are at the forefront of the field. Furthermore, a public database is constructed using LIDAR sensor data from diverse dusty environments. The database is made public for use in the training and benchmarking of dust filtering methods.

## 1. Introduction

LIDAR (LIght Detection And Ranging) sensors are used in mining and mineral processing to provide detailed three-dimensional measurements of the environment. These measurements are used for topographic mapping, rock characterization, autonomous mining vehicle navigation, and obstacle detection, among other applications.

However, mining operations such as blasting, crushing, secondary reduction, and material transport by earthmoving machines and trucks generate dust that can remain suspended in the air for extended periods. Dust particles scatter and absorb light, which introduces noise into LIDAR measurements [1]. This phenomenon can generate false measurements, producing erroneous distance readings, false detections, or missed detections of objects of interest [2]. To address this problem, dust measurements must be filtered from LIDAR readings [3].

Thus, using filtering algorithms allows for identification of dust signals and separation of them from other environmental data. Most algorithms model dust measurements as outliers and filter them by comparing the position of each measurement with those of its neighbors and/or by analyzing the intensity of the measurement. This is because dust measurements typically have a lower intensity than non-dust measurements. Moreover, new hyperspectral LIDARs can simultaneously obtain spatial and spectral information [4,5], meaning that each measurement is extended with spectral information. The inclusion of such information allows for a richer description of points and the possibility of using filtering algorithms that use these properties. However, given that these sensors are not yet widely adopted in standard mining applications such as obstacle detection and autonomous navigation, they are not included in this work. Therefore, our focus is on filtering methods that can be used in standard industrial LIDARs, such as those used in mining vehicles.

Among other techniques, filtering based on the use of machine learning techniques stands out because statistical classifiers can learn to identify patterns in data [6]. Statistical classifiers can be trained using different types of sensors and information sources, allowing them to adapt to various operating conditions in mining environments. They can significantly improve the accuracy of the 3D data obtained by effectively identifying and differentiating dust measurements.

Two challenges for the use of statistical classifiers in dust filtering are the processing speed and the availability of data for the training of models [3]. Real-time dust filtering is crucial in some applications, such as autonomous navigation and obstacle detection, which require real-time decision-making. Regarding the availability of training data, a related requirement is the availability of open dust databases to develop and benchmark different dust filtering methods.

In this paper, we address both challenges. First, we propose a neural network-based method capable of filtering dust measurements in real time from point clouds obtained using LIDARs. We train and validate the proposed method using real data. Second, we build a database using LIDAR sensor data from different dusty environments. This database is made public for use in the training and benchmarking of dust-filtering methods.

The main contributions of this paper are as follows: (1) a neural based method for the real-time dust filtering of point clouds obtained with LIDAR sensors, which includes a novel neural network encoding and processing architecture, as well as the use of novel features; (2) UCHILE-Dust, a database for training and testing dust filtering methods, which is made public.

## 2. Related Work

### 2.1. Traditional Dust Filtering Methods

Traditional dust filtering methods process each element of the point cloud iteratively and filter them considering the local density, the intensity of the measurement, or both.

**Statistical Outlier Removal (SOR).** Dust filtering is implemented by modeling dust measurement as outliers. For each point of the point cloud, the average distance $d_{i}$ to a neighborhood of *K* points is calculated and then compared to a threshold value *T* given by [3]

(1) T=μ±β·σ

where μ and σ are the mean and standard deviation of $d_{i}$, respectively, and β is a constant to be defined. If the average distance exceeds *T*, the point is considered an outlier and is filtered.

**Radius Outlier Removal (ROR).** In this method, the number of points, $n_{i}$, within a sphere with a radius of *R* centered on each point, $p_{i}$, is calculated [3]. If $n_{i}$ is smaller than a threshold value *N*, the point under analysis is considered an outlier. *R* and *N* are hyperparameters to be determined.

**Dynamic Radius Outlier Removal (DROR).** The ROR method tends to fail with LIDAR sensors because the use of a fixed radius cannot handle the variable resolution of the point cloud. In order to address this, DROR [7] proposes using a dynamic radius, $R_{dyn}$, which depends on the distance of the point and the angular resolution of the sensor. For points closer to the source, a fixed radius $R_{min}$ is used; otherwise, the radius is calculated as follows:

(2) $$R_{dyn}=\phi \cdot \alpha \cdot \sqrt{x^{2}+y^{2}}$$

where ϕ is a constant, α is the angular resolution of the LiDAR sensor, and (x,y) are the Cartesian coordinates of the point under analysis.

**Low-Intensity Outlier Removal (LIOR).** This method takes into account the fact that the measurements corresponding to the dust have a low intensity [8]. Based on this idea, the LIOR method [9] applies ROR filtering to points whose intensity is lower than a threshold μ. Points whose intensity is greater than μ are not filtered.

**Low-Intensity Dynamic Radius Outlier Removal (LIDROR).** The method proposed in [10] improves LIOR by implementing DROR filtering instead of ROR filtering, which means that a variable radius is used for filtering.

According to [3], LIDROR performs better than the SOR, ROR, DROR, and LIOR methods when it comes to dust filtration.

### 2.2. Machine Learning-Based Dust-Filtering Methods

The basic idea of these methods is to use a statistical classifier to determine if a point (measurement), or the points contained in a voxel, correspond to dust or not. Methods that use features computed on the voxels calculated from the point cloud are called *voxel-wise* methods, while methods that use the points of the point cloud directly are called *point-wise* methods. Naturally, point-wise methods are preferred because they are able to filter individual dust measurements.

Any statistical classifier can be used to perform the point-wise/voxel-wise classification, although only the use of Support Vector Machines (SVMs), Random Forests (RFs), and Neural Networks (NNs) has been reported in dust-filtering [2,3,6,11]. Regarding the employed features, the voxel-wise methods use the mean intensity and standard deviation of the points inside each voxel, as well as appearance features that characterize the type of material that reflect the sensors’ measurements. Some of the most commonly used appearance features are roughness [2,3,6], slope [2,3], planarity [3,6], and curvature [3,6]. These features are calculated from the eigenvalues obtained after performing a PCA projection of the voxel points.

In ref. [2], voxel-wise classification was implemented using SVM, RF, and NN classifiers. As features, mean intensity, standard deviation, roughness, and slope were used. The neural network classifier obtained the best results. In ref. [3], voxel-wise classification was also implemented using the same classifiers. For SVM and RF classifiers, the mean intensity, standard deviation, slope, and roughness were used as features, whereas for the NN classifier, the mean intensity, standard deviation, planarity, curvature, and the third eigenvalue were employed. The best results were obtained by the RF and NN classifiers. In ref. [6], a CNN (Convolutional Neural Network) was used to implement a voxel-wise classification using as features mean intensity, standard deviation, roughness, planarity, and curvature.

In ref. [11], point-wise classification was implemented using a U-net (an encoder–decoder type of CNN architecture), which required the point cloud to be transformed into a 2D LIDAR image. In this image, each pixel corresponds to a LIDAR measurement, and the rows and columns correspond to the vertical and horizontal angles of the measurement, respectively. Given that a multiecho LIDAR was used, the inputs to the U-net were the range and intensity values for the first echo and the last echo in each image position. The authors compare the proposed point-wise architecture with a voxel-wise architecture implemented using a standard CNN. The proposed point-wise architecture obtained slightly better results than the voxel-wise one.

The main drawback of the reported methods based on the use of statistical classifiers is that most of them employ a voxel-wise classification, which does not allow for the filtering of single measurements corresponding to dust; instead, voxels are classified as dust. This makes it hard to use these methods in cases where small details need to be determined (e.g., the characterization of small structures or the detection of small obstacles from a mobile vehicle). In the case of using a point-wise classification [11], the 2D projection of the point cloud lost 3D spatial information.

Therefore, there is a need for point-wise architectures for dust filtering that can fully utilize the 3D information contained in the point cloud. In the following section, we present a neural architecture capable of performing this kind of processing.

## 3. Proposed Dust-Filtering Method

The proposed dust-filtering method is based on the use of a modified version of the PointNet++ architecture [12], which receives point cloud data directly, groups the points hierarchically, computes internal feature representations, and classifies each point as dust or non-dust. For each LIDAR measurement, its 3D position, intensity, and temporal displacement are provided to the network. In the case where the data scan is acquired from a mobile vehicle, odometry information is also considered. Thus, the dust-filtering process takes into account geometric, intensity, and temporal features.

### 3.1. Neural Architecture: Reduced-PointNet++

The PointNet network, proposed in [13], is able to directly process the spatial coordinates and other attributes of the points to classify and segment a point cloud. Unlike previous approaches that required converting the point clouds into 2D or volumetric representations, PointNet works directly on 3D data without the need for voxelization or projections, which makes it more efficient in terms of memory and processing. PointNet is based on the use of symmetric functions, such as global max-pooling, which allows it to be invariant to the order of the points, a fundamental property in point clouds where there is no predefined order. In addition, the model incorporates learned transformations that align the point cloud before processing, reducing the variability introduced by differences in data orientation. However, due to its reliance on global feature aggregation, PointNet is limited in its ability to capture local relationships between points, which may affect its performance on tasks that require fine-structure information, as in the case of filtering dust measurements.

PointNet++, proposed in [12], improves PointNet by using hierarchical groupings of PointNet points and subnetworks on multiple scales, allowing greater capture of local features and spatial relationships. This modification allowed its use for dust filtering.

In order to achieve real-time processing of large point clouds, a reduced version of PointNet++ was designed in this work. Two big changes were made to PointNet++: first, the number of sampled points was reduced, which also reduced the number of abstraction layers (SA), and some blocks were eliminated to make the architecture simpler. Second, the MLPs configurations in both the abstraction and propagation blocks were adjusted. This led to a significant decrease in the number of parameters and an improvement in the efficiency of the model. Table 1 shows these changes. The reduced architecture was obtained empirically through iterative pruning. Layers were progressively removed while monitoring accuracy, and the process was repeated until a non-negligible degradation in performance was observed.

In Section 4, a comparison of the performance of PointNet++ and its reduced version is presented in dust-filtering tasks.

### 3.2. Point Cloud Features

Let us consider a point cloud $P^{t}$ containing points $p_{i}^{t}$ belonging to frame *t*, where *t* represents an instant in a temporal sequence of length *T*. Each point $p_{i}^{t}$ is defined by

(3) $$p_{i}^{t}=\left[c_{i}^{t},f_{i}^{t}\right],$$

where $c_{i}^{t}=\left(x_{i}^{t},y_{i}^{t},z_{i}^{t}\right)$ corresponds to spatial data and $f_{i}^{t}$ represents a vector of associated attributes, such as intensity ($g_{i}^{t}$), or other properties such as temporal information.

A first alternative to incorporating temporal information is to find the spatial differences between points in consecutive point clouds. For each point $p_{i}^{t}$ in $P^{t}$, we look for the nearest point in $P^{t-1}$. This is achieved by minimizing the following Euclidean distance:

(4) $$d_{ij}^{t-1,t}=∥c_{i}^{t}-c_{j}^{t-1}∥=\sqrt{{\left(x_{i}^{t}-x_{j}^{t-1}\right)}^{2}+{\left(y_{i}^{t}-y_{j}^{t-1}\right)}^{2}+{\left(z_{i}^{t}-z_{j}^{t-1}\right)}^{2}}.$$

Then, the nearest point $p_{j^{*}}^{t-1}$ is determined as

(5) $$j^{*}=\arg\min_{j}d_{ij}^{t-1,t},$$

and the spatial difference vector is computed as

(6) $$\Delta c_{i}^{t}=c_{i}^{t}-c_{j^{*}}^{t-1}.$$

The vector of spatial differences $\Delta c_{i}^{t}$, or its magnitude $m_{i}^{t}$, can then be used as a temporal attribute of each point.

A second alternative to incorporate temporal information, proposed in [14] as *temporal variation-aware interpolation*, is to generate an interpolated feature to represent the local information of the previous point cloud $P^{t-1}$ projected in the current point cloud $P^{t}$. To achieve this, first, for each point $p_{i}^{t}$ in $P^{t}$, we calculate the distances $d_{ij}^{t-1,t}$ to the *K*-nearest neighbors in $P^{t-1}$. Then, the interpolation weights for each neighbor point are computed as follows:

(7) $$w_{ij}^{t-1,t}=\beta \cdot \left(\alpha -\min(d_{ij}^{t-1,t},\alpha )\right).$$

with $d_{ij}^{t-1,t}$ defined by Equation (4) and α and β hyperparameters.

The weights are then normalized using the *softmax* function:

(8) $${\hat{w}}_{ij}^{t-1,t}=\frac{\exp(w_{ij}^{t-1,t})}{\sum_{k=1}^{K}\exp\left(w_{ik}^{t-1,t}\right)}.$$

Then, for each of the nearest neighbor points of $p_{i}^{t}$ in the previous point cloud, the intensity value and the differences of the intensity values are fed to an MLP layer with ReLU activation, and intermediate features are computed (see details of the network architecture in [14]):

(9) $$v_{ij}^{t-1,t}=\mathrm{ReLU}\left(\mathrm{MLP}\left(g_{j}^{t-1},g_{i}^{t}-g_{j}^{t-1}\right)\right),$$

Afterward, all intermediate features are agregated to generate the interpolated feature $h_{i}^{t}$ as

(10) $$h_{i}^{t}=\sum_{j=1}^{K}{\hat{w}}_{ij}^{t-1,t}⊙v_{ij}^{t-1,t}.$$

where ⊙ is an elemental multiplication.

As shown in Table 2, different variants of our dust-filtering method can be built depending on the information (feature vector) used:

- SI: Spatial + Intensity features.
- STdm: Spatial + Temporal-magnitude-difference features.
- STdv: Spatial + Temporal-vector-difference features.
- STi: Spatial + Temporal-interpolated features.
- SITdm: Spatial + Intensity + Temporal-magnitude-difference features.
- SITdv: Spatial + Intensity + Temporal-vector-difference features.
- SITi: Spatial + Intensity + Temporal-interpolated features.

Finally, in the case where the 3D data are acquired from a LIDAR mounted on a moving vehicle or robot, the odometry information is used to align the point clouds before the temporal features are computed. Thus, before calculating temporal features between point clouds $P^{t-1}$ and $P^{t}$, the points of $P^{t-1}$ are projected to *t* using the rotation and translation matrices between t−1 and *t*, $R_{t}$ and $T_{t}$, respectively. These matrices are calculated from the vehicle’s odometry.

## 4. Experimental Results

### 4.1. UCHILE-Dust Database

The UCHILE-Dust dataset was acquired during campaigns conducted from **September 2024 to February 2025**.

The OS0 was configured at **1024 × 64** resolution, providing a **360°** azimuth field of view, and operated in **dual-return** mode at **10 Hz**. Each frame was extracted with 3D coordinates and return intensity, and robot odometry was associated where applicable.

Recordings were saved either as PCAP files (direct OS0 stream) or ROS bag files (robot-mounted OS0 with odometry). A multistep preprocessing pipeline was applied, including frame extraction, odometry alignment (moving sensor case), multiecho merging, distance filtering, pre-labeling with dust-free references, manual annotation using *labelCloud*, and final conversion to the S3DIS format (Stanford Large-Scale 3D Indoor Spaces). The database is available at https://github.com/nicolasCruzW21/UCHILE-Dust, accessed on 4 September 2025. Table 3 shows a general overview of the database, considering the different subsets captured in different environments. It is important to note that the percentage of dust points in all subsets is less than 12%.

#### 4.1.1. Interior 1 and 2 Subsets

Captured indoors at the Field Robotics Laboratory of the Advanced Mining Technology Center (AMTC) of the Universidad de Chile (UCHILE) using a static OS0 LiDAR sensor. Dust was manually dispersed across the scene, which includes a combination of glass and concrete surfaces that introduce complexities with transparent and reflective materials. A rock breaker hammer is present in the center of the room.

#### 4.1.2. Exterior 1 and 2 Subsets

Captured outdoors in the AMTC courtyard. Dust was dispersed between the sensor and a nearby wall or in an open space to assess the impact of multipath reflections.

#### 4.1.3. Carén Subset

Captured in a large, dry, and windy open field with flat terrain and a quarry. The site is located in the Carén park in the Metropolitan Region of Chile and belongs to UCHILE. A Panther robot equipped with an OS0 LiDAR was used to collect data while in motion. Dust was introduced using an air blower. Carén is the most realistic subset for mobile perception tasks.

**Figure 1:**
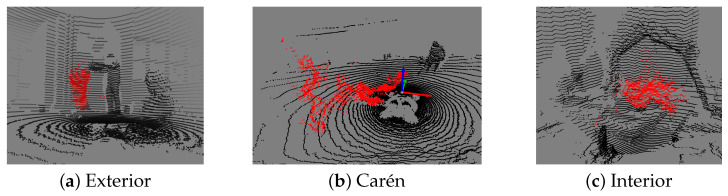
Example frames from the UCHILE-Dust dataset from Exterior (**a**), Carén (**b**), and Interior (**c**) subsets.

Example point cloud frames for each subset are shown in Figure 1.

### 4.2. Experimental Setup

Each variant of the model (SI, STdm, STdv, STi, SITdm, SITdv, and SITi) was trained using its respective training and validation sets. Three different learning rates were used for each variant: 0.01, 0.005, and 0.008. The learning rate that produced the best results was selected. This was done because the average accuracy varied depending on the learning rate used in each experiment. In all cases, a batch size of 16 was used. Other hyperparameters used are the following:

- The CrossEntropyLoss was used as a loss function but considering weights for the classes, since they are unbalanced. This weight consisted of the inverse of the proportion of each class within the corresponding training dataset.
- The number of training epochs was set at 100, but Early Stopping was implemented with 10 epochs of *patience* relative to the average accuracy value in the validation set. This metric was chosen as it is invariant to class imbalance.
- Data Augmentation methods were used: rotations, scaling, occlusion, and noise.
- A dropout rate of 0.7 was used to reduce overfitting and was applied to the last convolution layer before the classification layers.

We used two methods as a baseline: LIDROR, the traditional method with the best reported dust-filtering results, and the CNN-based method [6], which reports better filtering results than LIDROR in [6]. For the CNN-based method, we used the hyperparameters in the original paper and learning rate values of 0.01, 0.005, and 0.008. The learning rate that produced the best results was selected. For LIDROR, we determined the hyperparameters using grid search and the following ranges: μ∈{90,100,110}, R∈{0.001,0.01}, ϕ∈{0.01,0.05,0.1}, and N∈{4,6,8}. Table 4 shows the parameters obtained. The parameter α is the angular resolution of the sensor, it depends on the LIDAR model and was set to 0.006134.

The following metrics were employed to assess the quality of the various methods employed for dust classification: accuracy, precision, recall, and F1-score. Accuracy is the proportion of all classifications that are correct, precision is the proportion of dust classifications that are actually dust, recall is the proportion of actual dust samples that are correctly classified as dust, and F1-score is the harmonic mean of precision and recall.

### 4.3. Results in Real Environments with Static Sensors

The results of applying different dust-filtering methods to the Interior 1, Interior 2, Exterior 1, and Exterior 2 datasets are shown in Table 5, Table 6, Table 7 and Table 8, respectively. In each instance, the methods are trained using exclusively data from the corresponding datasets.

The results obtained allow for the following conclusions to be drawn. All variants of the proposed method demonstrate superior performance compared to the LIDROR and CNN methods, with the exception of the Interior 1 dataset. The CNN method consistently performs better than LIDROR, and in the Interior 1 dataset, it shows the best performance. In the Interior 2, Exterior 1, and Exterior 2 datasets, one of the variants of the proposed methods achieves the best performance. In the Exterior 1 dataset, both baselines have very low precision: 0.03 for LIDROR and 0.09 for CNN. This means that they are unable to filter dust in these cases.

The most challenging datasets are Interior 1 and Exterior 2. In these datasets, all methods have low precision. Given that in deployment in a real environment false positives can be costly, this dataset represents an ideal benchmark for further experimentation. Furthermore, low F1-Scores are expected in these kinds of datasets and are driven by the large imbalance between classes.

Finally, a comparison of the proposed variants reveals that the utilization of both temporal difference and intensity features is generally superior to the application of either temporal differences or intensity features alone.

### 4.4. Results in Real Environments with Moving Sensors

The results of applying different dust-filtering methods to the Carén dataset, in which the LIDAR sensor is mounted on a mobile robot, are shown in Table 9 and Table 10. Table 9 shows the results when the odometry correction is not used, and Table 10 shows the results when the odometry correction is used.

The results obtained allow for the following conclusions to be drawn. All variants of the proposed method outperform both baselines. In fact, LIDROR achieves very low precision with this dataset. The CNN-based method also shows lower precision values than most variants of the proposed method.

Secondly, as demonstrated in the preceding subsection, the utilization of temporal features has been shown to enhance the efficacy of dust filtering. However, the alignment and analysis of the geometric characteristics of LIDAR points belonging to consecutive frames is challenging when utilizing moving sensors. This is because the points are in different reference systems. In static scenes, temporal variations arise predominantly from dust motion; therefore, magnitude-based temporal descriptors combined with intensity (e.g., SITdm) are particularly effective. When odometry is not applied, the ego-motion of the platform makes the entire environment appear to move, introducing apparent motion in all points; under these conditions, purely geometric temporal cues become less reliable, and intensity-driven temporal interpolation (e.g., STi/SITi) becomes more informative. Once odometry correction is applied, consecutive frames are geometrically aligned, compensating for global motion and revealing the true local dynamics of dust; in this case, directional temporal descriptors combined with intensity (e.g., SITdv) better capture the irregular, locally dispersed motion of dust compared to the coherent behavior of static surfaces. Therefore, we hypothesize that the application of odometry correction and temporal features allows for a better characterization and filtering of dust points. This phenomenon is evident in Table 10, where the results of the proposed variants demonstrate enhancement when odometry correction is used. The only two cases in which the accuracy decreases are those corresponding to the STi and STdv variants. The SITdv variant yielded the best results, with a precision value of 0.7, a recall of 0.97, and an F1-score of 0.82.

**Figure 2:**
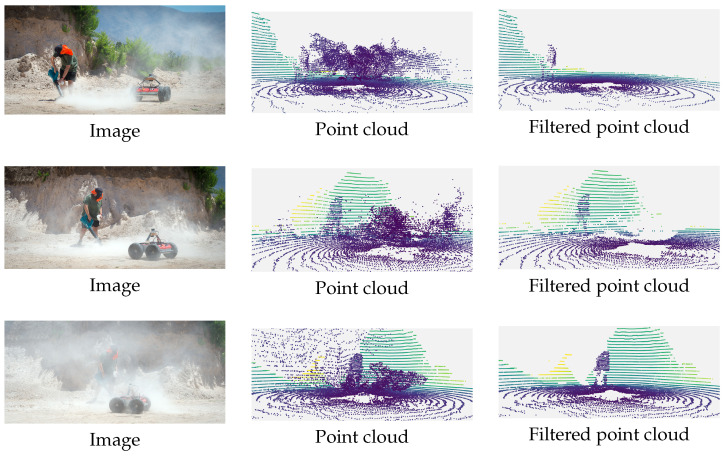
Comparison of original images, point clouds with dust, and filtered point clouds without dust.

Figure 2 shows three examples of the dust filtering method in action. In all three cases, it can be seen that the method removes most of the observed dust.

### 4.5. Measuring the Generalization Capabilities of the Method

To evaluate the generalization capabilities of the variants of the proposed methods and the CNN method, the methods were trained and validated using the Interior 1, Interior 2, Exterior 1, and Exterior 2 datasets, and then tested using the Carén dataset. The results of these experiments are shown in Table 11 and Table 12, which present the results for cases with and without odometry.

Upon comparing the results of the proposed methods trained and validated using the Carén dataset (Table 9 and Table 10) reveals that there is only a slight advantage to in-domain training relative to training in one environment and testing in another (Table 11 and Table 12). This finding indicates that the model is learning underlying dust features rather than environment-specific features, such as the relative position of the objects in the scene. This finding suggests that the model is generalizing correctly.

In the case of the CNN-based method, we observe a slightly higher drop in precision compared to the proposed methods. In all cases, the proposed methods achieve higher precision, recall, and F1-score.

Another conclusion is that the benefits of using odometry correction are lost in this experimental setup. In most cases, no improvement in results is observed. One possible reason for this is that the training and validation sets do not account for odometry correction.

### 4.6. Performance Comparison Between PointNet++ and Reduced-PointNet++

As a final experiment, a comparison was carried out between using the original PointNet++ architecture and the reduced-PointNet++ architecture proposed here for all of the methods presented in this work.

All experiments were carried out on a platform with a 12 GB VRAM NVIDIA GTX TITAN GPU (NVIDIA, Santa Clara, CA, USA), a 12-core Intel^®^ Core™ i7-8700K processor (Intel, Santa Clara, CA, USA) running at 3.70 GHz, and 16 GB of RAM. Table 13 shows a comparison of the execution times of both architectures. It can be seen that the reduced-PointNet++ architecture outperforms the original PointNet++ architecture in terms of speed. The execution times are, on average, 50% shorter.

It must be noted that this reduction in execution time does not sacrifice performance. Table 14 shows the results of using the original PointNet++ architecture when the methods are trained on the Interior 1, Interior 2, Exterior 1, and Exterior 2 datasets but tested on the Carén dataset, without using odometry information. These results are directly comparable to those in Table 11. Comparing both tables reveals that the results are similar. In some cases, the original PointNet++ obtains slightly better results, while in others, the proposed reduced-PointNet++ obtains slightly better results.

These results show that reducing the network size is a valid approach for the dust detection problem, but it also shows that there is still room for further prunning since the reduction in parameters did not affect performance.

## 5. Discussion

### 5.1. Analysis of the Results

This work presents a real-time dust filtering method for LIDAR point clouds based on a reduced-PointNet++ architecture. From the results obtained, it is clear that the proposed approach outperforms the baseline heuristic method and the baseline machine learning method. Furthermore, the experiments demonstrated that temporal information is useful in distinguishing dust from static objects, and the integration of odometry further improved the performance in mobile applications.

This is a promising approach for robotic applications in challenging dust-filled environments, since it allows filtering of dust from LiDAR measurements in order to perform basic navigation tasks such as SLAM, object avoidance, and emergency stops. Furthermore, the proposed reduction in the network size, which in turn reduces the computational requirements and inference times, allows the network to be deployed in embedded systems such as those found in robotic platforms, which are usually constrained by restrictions such as size and, for dusty environments, passive cooling.

The approach was tested and developed using the UCHILE-Dust dataset, which is made publicly available to support the development and benchmarking of future methods. The dataset includes indoor, outdoor, and mobile robot recordings, covering a wide range of scenarios with varying dust densities, allowing for a wide range of possible applications. Our experiments showed that models trained on UCHILE-Dust generalized well from static environments to mobile robot deployments. This suggests that models trained on this dataset learn dust-specific features rather than environment-specific features such as the relative positions of the objects in the scene (layout).

### 5.2. Mining Applications

2D and 3D LIDAR sensors are used in mining and mineral processing to provide detailed three-dimensional measurements of the environment. Given that their use can be hindered by the presence of dust generated in different mining processes (blasting, crushing, secondary reduction, material transport by earthmoving machines and trucks, etc.), it seems natural to use dust-filtering methods to reduce the dust from LIDAR observations.

For example, dust filtering can be used to increase the robustness of obstacle detection and autonomous navigation systems in haul trucks in open pit mines. It can also be used to filter transient dust clouds after blasting, to increase the accuracy of volume calculations in stockpile volumetrics, to improve 3D measurements and rock detection in impact hammer automation, and to remove noisy measurements that can produce false deformation alerts in pit wall monitoring, among other applications.

In our case, we are applying the proposed method to our autonomous navigation and loading system using LHD (load–haul–dump) vehicles [15] and to our autonomous impact hammer operating system [16]. We expect field data from these deployments to be available in the near future to assess and report on the benefits of the dust-filtering method in these two applications.

## 6. Conclusions

This article proposes a dust-filtering method based on the use of a neural network. This method can filter dust measurements from point clouds obtained by using LIDARs in real time. The method was validated using real data, yielding results at the forefront of the field. In addition, a public database was created using LIDAR sensor data from various dusty environments. This database is expected to play a crucial role in the development and validation of dust filtering methods.

Future work will include measuring the advantages of using this type of algorithm in mining applications with LIDAR data. These applications include detecting obstacles from mining vehicles in open pits, autonomously navigating in mining tunnels, autonomous operation of impact hammers, and creating 3D models of stockpiles, among others.

Furthermore, it is worthwhile to study how novel hyperspectral LIDARs can be applied in mining applications.

## Figures and Tables

**Table 1:** Comparison of key blocks of PointNet++ and reduced-PointNet++, highlighting the modifications in the number of sampled points and the configuration of the MLPs.

Block	PointNet++	Reduced-PointNet++
SA1	1024 pts, r = 0.1, 32 nbr, MLP: [32, 32, 64]	512 pts, r = 0.1, 32 nbr, MLP: [16, 16, 32]
SA2	256 pts, r = 0.2, 32 nbr, MLP: [64, 64, 128]	128 pts, r = 0.2, 32 nbr, MLP: [32, 32, 64]
SA3	64 pts, r = 0.4, 32 nbr, MLP: [128, 128, 256]	–
SA4	16 pts, r = 0.8, 32 nbr, MLP: [256, 256, 512]	–
FP4	in_ch: 768, MLP: [256, 256]	–
FP3	in_ch: 384, MLP: [256, 256]	–
FP2	in_ch: 320, MLP: [256, 128]	in_ch: 96, MLP: [64, 32]
FP1	in_ch: 128, MLP: [128, 128, 128]	in_ch: (32 + num_feats), MLP: [32, 32]
Conv1d	128 → 128	32 → 32
BatchNorm1d	128	32
Dropout	*p* = 0.7	*p* = 0.7
Conv1d (final)	128 → num_classes	32 → num_classes

**Table 2:** Variants of the proposed dust filtering method and the features used in each case. For simplicity in the notation, the temporal indices *t* are omitted.

Variant	Features Vector
SI	$\left[x_{i},y_{i},z_{i},g_{i}\right]$
STdm	$\left[x_{i},y_{i},z_{i},m_{i}\right]$
STdv	$\left[x_{i},y_{i},z_{i},\Delta c_{i}\right]$
STi	$\left[x_{i},y_{i},z_{i},h_{i}\right]$
SITdm	$\left[x_{i},y_{i},z_{i},g_{i},m_{i}\right]$
SITdv	$\left[x_{i},y_{i},z_{i},g_{i},\Delta c_{i}\right]$
SITi	$\left[x_{i},y_{i},z_{i},g_{i},h_{i}\right]$

**Table 3:** Overview of the UCHILE-Dust dataset subsets, including number of recordings, number of point clouds, total number of points, percentage of dust points, data format, and train/val/test split percentages.

Subset	Recordings	Point Clouds	Points	% Dust	Format	Train/Val/Test (%)
Interior 1	10	1874	72,741,326	4.1%	PCAP	82/09/09
Interior 2	12	1740	71,225,483	11.2%	PCAP	70/15/15
Exterior 1	10	1529	45,311,889	3.2%	PCAP	84/08/08
Exterior 2	13	1885	75,820,483	3.6%	PCAP	66/17/17
Carén	13	7089	234,929,532	8.1%	Rosbag	46/26/28

**Table 4:** Parameters used for the LIDROR method.

Subset	μ	$R_{\min}$	ϕ	N
Interior 1	90	0.001	0.01	4
Interior 2	90	0.001	0.01	4
Exterior 1	90	0.010	0.01	4
Exterior 2	90	0.010	0.01	4
Carén	90	0.001	0.01	4

**Table 5:** Results of dust filtering using different methods in the Interior 1 dataset.

Method	Learn. Rate	Accuracy (Avg)	Precision (Dust)	Recall (Dust)	F1-Score (Dust)
SI	0.008	0.79	0.12	0.93	0.22
STdm	0.008	0.87	0.17	1.00	0.29
STdv	0.008	0.86	0.17	0.99	0.29
STi	0.010	0.89	0.26	0.91	0.41
SITdm	0.010	0.86	0.17	0.97	0.29
SITdv	0.005	0.77	0.13	0.85	0.23
SITi	0.005	0.80	0.15	0.85	0.26
LIDROR	–	0.76	0.14	0.77	0.24
CNN	0.008	0.86	0.55	0.77	0.64

**Table 6:** Results of dust filtering using different methods in the Interior 2 dataset.

Method	Learn. Rate	Accuracy (Avg)	Precision (Dust)	Recall (Dust)	F1-Score (Dust)
SI	0.008	0.94	0.53	0.94	0.68
STdm	0.008	0.92	0.60	0.88	0.71
STdv	0.010	0.84	0.50	0.74	0.59
STi	0.008	0.87	0.55	0.80	0.65
SITdm	0.008	0.94	0.68	0.91	0.78
SITdv	0.008	0.95	0.66	0.93	0.77
SITi	0.008	0.94	0.60	0.92	0.73
LIDROR	–	0.86	0.21	0.96	0.35
CNN	0.005	0.95	0.55	0.98	0.71

**Table 7:** Results of dust filtering using different methods in the Exterior 1 dataset.

Method	Learn. Rate	Accuracy (Avg)	Precision (Dust)	Recall (Dust)	F1-Score (Dust)
SI	0.010	0.99	0.48	0.99	0.65
STdm	0.005	0.99	0.35	0.98	0.51
STdv	0.005	0.99	0.27	0.99	0.42
STi	0.005	0.97	0.46	0.95	0.62
SITdm	0.010	0.99	0.68	0.99	0.81
SITdv	0.005	0.99	0.54	0.98	0.70
SITi	0.010	0.99	0.51	0.98	0.67
LIDROR	–	0.91	0.03	0.98	0.06
CNN	0.005	0.84	0.09	0.93	0.16

**Table 8:** Results of dust filtering using different methods in the Exterior 2 dataset.

Method	Learn. Rate	Accuracy (Avg)	Precision (Dust)	Recall (Dust)	F1-Score (Dust)
SI	0.005	0.98	0.38	0.99	0.55
STdm	0.010	0.98	0.35	0.99	0.52
STdv	0.010	0.97	0.36	0.97	0.53
STi	0.010	0.94	0.20	0.97	0.34
SITdm	0.005	0.98	0.38	0.99	0.55
SITdv	0.005	0.98	0.39	0.99	0.57
SITi	0.008	0.98	0.44	0.99	0.61
LIDROR	–	0.88	0.09	0.98	0.16
CNN	0.008	0.85	0.32	0.89	0.47

**Table 9:** Results of dust filtering using different methods in the Carén dataset (without odometry correction).

Method	Learn. Rate	Accuracy (Avg)	Precision (Dust)	Recall (Dust)	F1-Score (Dust)
SI	0.005	0.98	0.61	0.98	0.75
STdm	0.010	0.95	0.43	0.96	0.60
STdv	0.010	0.95	0.47	0.94	0.62
STi	0.008	0.96	0.72	0.94	0.81
SITdm	0.010	0.98	0.60	0.98	0.74
SITdv	0.008	0.98	0.62	0.98	0.76
SITi	0.005	0.98	0.63	0.97	0.76
LIDROR	–	0.89	0.18	0.98	0.30
CNN	0.01	0.94	0.46	0.94	0.62

**Table 10:** Results of dust filtering using different methods in the Carén dataset (with odometry correction).

Method	Learn. Rate	Accuracy (Avg)	Precision (Dust)	Recall (Dust)	F1-Score (Dust)
SI	0.005	0.98	0.61	0.98	0.75
STdm	0.008	0.96	0.56	0.95	0.70
STdv	0.008	0.95	0.41	0.96	0.58
STi	0.005	0.96	0.56	0.95	0.71
SITdm	0.005	0.98	0.64	0.98	0.77
SITdv	0.005	0.98	0.70	0.97	0.82
SITi	0.008	0.98	0.62	0.98	0.76
LIDROR	–	0.89	0.18	0.98	0.30
CNN	0.005	0.94	0.46	0.94	0.62

**Table 11:** Generalization results. Training on AMTC datasets and testing on the Carén dataset (without odometry correction).

Method	Learn. Rate	Accuracy (Avg)	Precision (Dust)	Recall (Dust)	F1-Score (Dust)
SI	0.005	0.98	0.58	0.98	0.73
STdm	0.008	0.96	0.46	0.95	0.62
STdv	0.005	0.95	0.41	0.95	0.57
STi	0.005	0.96	0.64	0.94	0.76
SITdm	0.005	0.98	0.60	0.98	0.74
SITdv	0.008	0.97	0.54	0.98	0.70
SITi	0.010	0.98	0.57	0.98	0.72
LIDROR	–	0.90	0.18	0.98	0.30
CNN	0.008	0.93	0.39	0.97	0.56

**Table 12:** Generalization results. Training on AMTC datasets and testing on the Carén dataset (with odometry correction).

Method	Learn. Rate	Accuracy (Avg)	Precision (Dust)	Recall (Dust)	F1-Score (Dust)
SI	0.008	0.98	0.56	0.98	0.71
STdm	0.008	0.96	0.50	0.96	0.66
STdv	0.005	0.96	0.49	0.95	0.65
STi	0.010	0.96	0.52	0.95	0.67
SITdm	0.005	0.98	0.60	0.98	0.75
SITdv	0.008	0.98	0.58	0.98	0.73
SITi	0.008	0.98	0.57	0.98	0.72
LIDROR	–	0.90	0.18	0.98	0.3
CNN	0.008	0.94	0.40	0.96	0.57

**Table 13:** Comparison of the execution times of PointNet++ versus reduced-PointNet++.

Method	PointNet++ (s)	Reduced-PointNet++ (s)
SI	0.1383	0.0676
STdm	0.1398	0.0672
STdv	0.1419	0.0679
STi	0.1420	0.0694
SITdm	0.1372	0.0710
SITdv	0.1435	0.0664
SITi	0.1435	0.0630
Average	0.1409	0.0675

**Table 14:** Generalization results obtained using the original PointNet++ architecture. Training on AMTC datasets and testing on the Carén dataset (without odometry correction).

Method	Learn. Rate	Accuracy (Avg)	Precision (Dust)	Recall (Dust)	F1-Score (Dust)
SI	0.005	0.98	0.55	0.98	0.71
STdm	0.005	0.96	0.46	0.96	0.62
STdv	0.005	0.95	0.51	0.94	0.66
STi	0.010	0.96	0.51	0.96	0.67
SITdm	0.005	0.98	0.58	0.98	0.73
SITdv	0.005	0.98	0.62	0.97	0.76
SITi	0.008	0.98	0.61	0.97	0.75

## Data Availability

The database is available at https://github.com/nicolasCruzW21/UCHILE-Dust, accessed on 4 September 2025.
